# Micronodular Basal Cell Carcinoma Presenting as an Achromic Macule

**DOI:** 10.7759/cureus.49806

**Published:** 2023-12-01

**Authors:** Jorge Alberto Cortez Vila, Rosa María Lacy Niebla, Leticia Boeta Ángeles

**Affiliations:** 1 Dermatology, Dr. Manuel Gea González General Hospital, Mexico City, MEX; 2 Dermatology, Hospital Juárez Centro, Mexico City, MEX

**Keywords:** basal cell carcinoma, morpheiform, micronodular, achromic, macule

## Abstract

A 63-year-old woman with light skin and a history of chronic sun damage presented with a painless, pale macule on her nasal tip. A punch biopsy was performed due to concerns about skin conditions like vitiligo or morpheaform basal cell carcinoma (BCC). The biopsy confirmed a micronodular BCC, an unusual presentation, as these typically manifest as an erythematous macule or thin papule/plaque. This report highlights the importance of considering various factors and differential diagnoses to ensure the best patient care and the need for vigilance in diagnosing rare presentations of BCC.

## Introduction

Basal cell carcinoma (BCC) is the most common skin malignancy, accounting for 80% of keratinocyte cancers [1]. Clinical presentations of this entity include nodular, superficial, infundibulocystic, fibroepithelial, morpheaform/sclerodermiform, and infiltrating BCC. There are additional descriptions according to their histopathological characteristics, such as micronodular, pigmented, and basosquamous variants. Each presents typical morphological characteristics that lead us to suspect their diagnosis [2]. In this study, we discuss the case of a patient with a micronodular BCC showing an atypical presentation as an achromic macule.

## Case presentation

**Figure 1:**
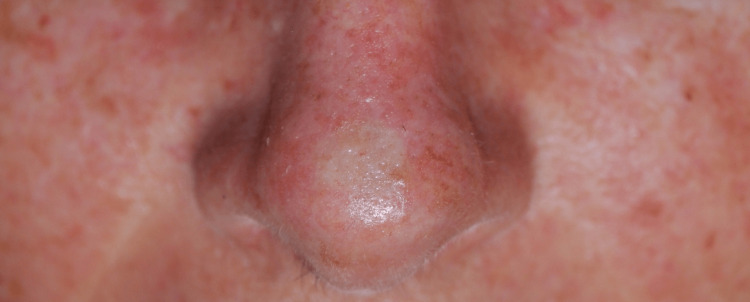
Achromic macule on the patient’s nasal tip.

A 63-year-old woman presented with an achromic macule on the nasal tip. During the physical examination, an asymptomatic, poorly defined achromic macule measuring 10 mm in diameter was observed, with no palpable neck lymphadenopathies detected (Figure 1). The lesion was noticed 1.5 months before the consultation. The patient reported no prior treatment and denied experiencing any additional symptoms related to the lesion. She had a family history of hepatic and breast malignancies and a personal clinical background of chronic photodamage, marked by multiple solar lentigines, fine wrinkles, actinic keratosis, some macular hypopigmentations, and seborrheic keratosis, on skin classified as Fitzpatrick type II.

**Figure 2:**
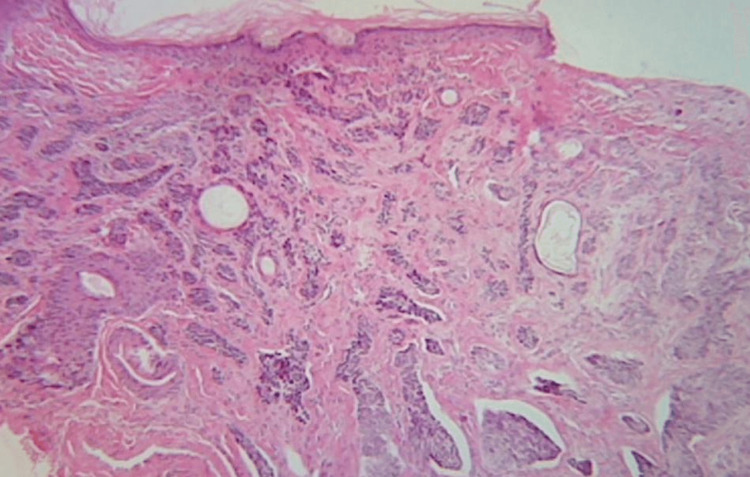
Histopathological features showing micronodular neoplastic basaloid cells aggregates (hematoxylin and eosin, x10).

An incisional 4 mm punch biopsy was carried out on the nasal tip due to the differential diagnoses of vitiligo, morpheaform BCC, or a hypopigmented macular lesion without further clinical significance. Histopathological examination showed hyperkeratosis with an atrophic epidermis and elastosis in the upper dermis. Small aggregates of neoplastic basaloid cells originating from the epidermis extended up to the mid-reticular dermis. The cells showed hyperchromatic large oval nucleus, moderate pleomorphism, mitosis, and individual necrosis. These aggregates were surrounded by a dense collagenous and fibromixoid stroma, exhibiting discrete refraction artifacts (Figure 2). These features were in line with the histologic pattern of a micronodular BCC.

Due to the topography, size, and aggressive nature of the BCC subtype, we performed Mohs micrographic surgery. Two stages were required for complete cancer removal. Subsequently, a frontonasal advancement flap was executed to close the surgical wound. There were no complications during the procedure. After a three-month postoperative period, the patient showed excellent healing, and no recurrence evidence has been noticed in a 10-year follow-up period.

## Discussion

The skin inspection and correlation with each patient's clinical history constitute the initial step in the diagnosis of BCC. The following are some crucial factors that must be examined: Fitzpatrick skin types I and II, age, light eyes, freckles, red hair, UV radiation exposure, and signs of chronic sun damage [1-3]. The patient's skin tone, along with the presence of skin photodamage featuring multiple solar lentigines and wrinkles, and the patient's age, led us to believe that the patient's lesion could be a morpheaform BCC, despite the initial suspicion of vitiligo based on the clinical appearance. The morpheaform BCC is characterized by an infiltrating plaque with poorly defined borders and a shiny surface that is usually encountered on the head and neck [2]. These features were not entirely consistent with the patient's lesion, but we believed that the achromic macule could be an indolent form of presentation.

To identify the kind of BCC and execute Mohs surgery to treat it, a punch biopsy of the lesion was performed. This was done as histopathologic examination is acknowledged as the reference standard in the diagnosis of BCC [3]. The biopsy revealed features that were consistent with a micronodular BCC rather than a morpheaform one.

The interesting aspect of this case is that the tumor clinically appeared more consistent with a vitiliginous macule or a morpheaform BCC, despite not being sclerous upon palpation, as opposed to a micronodular BCC. Micronodular BCCs often present as an erythematous macule, thin papule/plaque [2,4], or an elevated or flat yellow-whitish infiltrated tumor that rarely ulcerates. Moreover, they are commonly located on the skin of the back [5]. Therefore, a bibliographical review was conducted to search for other reports comparable to ours. During this review, we discovered a case report of a morpheaform BCC in a female patient. This case presented as a hypopigmented macule on the glabella and had been treated for years as vitiligo with topical steroids. The authors mention that this type of BCC can be misdiagnosed as various depigmented conditions [6]. With this case report, we emphasize that a micronodular BCC might resemble a vitiliginous lesion, underscoring the importance of maintaining a high level of suspicion in the diagnosis.

## Conclusions

These types of lesions should not be disregarded as insignificant. It is crucial to consider all external factors and potential hazards affecting the patients, assess various differential diagnoses, and eliminate those that could jeopardize the patient's quality of life or survival.

The possibility that a micronodular BCC could be presented as a hypopigmented macule should also be noted. To our knowledge, this would be a very infrequent clinical presentation.
